# Evaluation of effectiveness of treatment paradigm for newly diagnosed type 2 diabetes patients in Chin: A nationwide prospective cohort study

**DOI:** 10.1111/jdi.13092

**Published:** 2019-07-02

**Authors:** Xiaoling Cai, Dayi Hu, Changyu Pan, Guangwei Li, Juming Lu, Qiuhe Ji, Benli Su, Haoming Tian, Shen Qu, Jianping Weng, Danyi Zhang, Jie Xu, Linong Ji

**Affiliations:** ^1^ Departments of Endocrinology and Metabolism Peking University People's Hospital Beijing China; ^2^ Cardiology Peking University People's Hospital Beijing China; ^3^ Department of Endocrinology and Metabolism Chinese People's Liberation Army General Hospital Beijing China; ^4^ Department of Endocrinology and Metabolism Fuwai Hospital Beijing China; ^5^ Department of Endocrinology and Metabolism The Fourth Military Medical University Xi Jing Hospital Xi An China; ^6^ Department of Endocrinology and Metabolism The Second Affiliated Hospital Dalian Medical University Dalian China; ^7^ Department of Endocrinology and Metabolism Sichuan University West China Hospital Chengdu China; ^8^ Department of Endocrinology and Metabolism Shanghai Tenth People's Hospital Shanghai China; ^9^ Department of Endocrinology and Metabolism The Third Affiliated Hospital Sun Yat‐Sen University Guangzhou China; ^10^ VitalStrategic Research Institute Shanghai China

**Keywords:** Clinical effectiveness, Newly diagnosed, Type 2 diabetes

## Abstract

**Aims/Introduction:**

Data of nationwide glycemic control and hypoglycemic treatment patterns in newly diagnosed type 2 diabetes patients in China are absent. The aim of this study was to assess the evolution of treatment patterns for newly diagnosed type 2 diabetes patients and the clinical outcomes during 12‐month follow up.

**Materials and Methods:**

This is an observational prospective cohort study with 12 months of follow up. Patients with a diagnosis of type 2 diabetes for <6 months were enrolled. Glycated hemoglobin A1c (HbA1c) levels and hypoglycemic treatment patterns were collected at baseline and at every 3 months of follow up.

**Results:**

A total of 79 hospitals were recruited, consisting of 5,770 participants. The mean HbA1c was 8.4 ± 2.5% at baseline, and decreased to 6.7 ± 1.2% at 12 months with 68.5% of patients achieving HbA1c <7%. At baseline, 44.6% of the patients were without hypoglycemic medications, 37.7% had oral hypoglycemic agents and 17.7% received insulin treatment. Determinants of change in HbA1c were treatment patterns, comorbidities, baseline characteristics such as obesity and smoking, regions, and tiers of hospitals. Associated factors with treatment alterations were time of follow up, treatment patterns, patient‐reported reasons such as the economic factors and poor efficacy.

**Conclusions:**

In newly diagnosed type 2 diabetes patients, compared with patients without medications, patients with one oral hypoglycemic agent had higher possibilities of reaching glycemic control, whereas patients using insulin had lower possibilities of reaching the target. Factors associated with change in HbA1c and treatment alterations were also revealed.

## Graphical abstract

This is a prospective, nationwide multicenter, observational cohort study with 12‐month follow up, a study of the China Cardiometabolic Registry, with the aim to evaluate treatment patterns and the clinical outcomes for newly diagnosed type 2 diabetes patients in China, and to assess the associated factors with treatment changes during the 12‐month follow‐up period (CCMR‐NEW2D study).

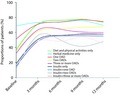

## Introduction

Type 2 diabetes is distinguished by hyperglycemia, insulin resistance, relative lack of insulin, and with micro‐ and macrovascular disease. Currently, China has the largest number of people with diabetes^1^, ^2^. The prevalence of diabetes increased rapidly to 11.6% in 2010 and 10.9% in 2013^3^, ^4^, with an estimated prevalence of 8.1% for newly detected diabetes, which has placed a huge economic burden on China. Therefore, effective clinical management of newly diagnosed type 2 diabetes mellitus patients is critically needed to attenuate disease progression and to reduce complications.

As recommended by the American Diabetes Association guideline^5^, ^6^ and the China Diabetes Society guideline^7^, ^8^, for newly diagnosed type 2 diabetes patients, oral hypoglycemic agents (OHAs) monotherapy or combination therapy, or in combination with insulin therapy are strategies that can be selected individually according to the patient's glycated hemoglobin A1c (HbA1c) level^5^, ^6^. Additionally, weight loss, risk of hypoglycemia and risks of side‐effects that might be caused by antidiabetes treatment should also be considered.

Although some clinical characteristics and treatment patterns in Chinese type 2 diabetes patients were reported previously^9^, ^10^, ^11^, ^12^, ^13^, ^14^, few data were available for newly diagnosed type 2 diabetes patients, and nationwide data have as yet not been collected. Therefore, we designed the present prospective, nationwide multicenter, observational cohort study with 12‐month follow up, a study of the China Cardiometabolic Registry (CCMR), with the aim to evaluate treatment patterns and the clinical outcomes for newly diagnosed type 2 diabetes patients in China, and to assess the associated factors with treatment changes during the 12‐month follow‐up period (CCMR‐NEW2D study).

## Methods

### Study design and population

The present study was a prospective, observational cohort study with a 12‐month follow‐up period. From June 2012 to February 2014, patients from 81 hospitals (community hospitals [tier 1], secondary/city level hospitals [tier 2] and teaching or comprehensive central hospitals [tier 3]) across six geographic regions of China (north, south, east, southwest, northeast, northwest) were recruited. Participants were enrolled at department of endocrinology and internal medicine clinics. The inclusion criteria were: (i) patients with aged ≥20 years; (ii) patients with confirmed diagnosis of type 2 diabetes according to the World Health Organization criteria, as recommended by the guideline of China Diabetes Society^7^, ^8^ within 6 months before screening; and (iii) patients who signed the consent form and were willing to return for all follow‐up visits. The exclusion criteria were: (i) patients who were pregnant or breast‐feeding or planned to be pregnant within 1 year; (ii) patients who were participating in another clinical trial; (iii) patients who were not willing to or not able to return to the same hospital every 3 months for the follow‐up visits after enrollment; and (iv) patients without clear information regarding the medication used. Ethical approval was first obtained from the ethics committees of Peking University People's Hospital and then was approved by all the participating hospitals. All patients signed the informed consent form before participation. The CCMR‐NEW2D study was registered at http://www.clinicaltrials.gov (NCT01525693).

### Study procedures and data collection

All patients received routine lifestyle suggestions, such as diet and exercise, by the investigators and also medications prescribed by the investigators. These patients were required to return to the same physician for the follow‐up visits at 3, 6, 9 and 12 months after the first visit. If the patient was lost to follow up, a structured telephone interview would be carried out by the investigator to ascertain the patient's condition. The definition for compliance was set as whether the patients routinely took medicines in accordance with the prescriptions, and if not, the specific reasons were required. For patients who were not treated with OHA or insulin, the definition for compliance was set as whether the patients routinely came back for visits.

At baseline and during the follow‐up period, the information as follows was to be collected from each patient: (i) demographics including age, sex, residential region, educational level and social‐economic status; (ii) diabetes and family histories; (iii) medical history, including any major medical procedure or surgery that occurred within 12 months; (iv) comorbidities, including hypertension, dyslipidemia, cardiovascular disease, diabetes‐related complications and cancer; (v) health behavior, including smoking history and exercise pattern; (vi) physical examinations and laboratory tests, including height, bodyweight, sitting blood pressure, fasting plasma glucose, HbA1c and fasting lipid profile; and (vii) adverse events and severe adverse effects. The self‐reported hypoglycemic questionnaire and self‐evaluated quality of life were also collected. The definition of occasional exercise was that patients exercised <150 min per week, as the recommendation of exercise by the Chinese Diabetes Guideline is that type 2 diabetes patients should have at least 150 min of exercise per week. Specific information about the hypoglycemic treatments was identified, including diet and physical activities only, use of herbal medicine only, use of OHAs (including metformin, sulfonylureas and glinides, α‐glucosidase inhibitor, thiazolidinediones and dipeptidyl peptidase‐4 inhibitors) and use of different types of insulin. The drugs’ name, dosage and daily times were all recorded in detail. According to the Chinese Diabetes Guideline^7^, ^8^, the body mass index (BMI) cut‐off values are categorized as BMI <19 is thin, BMI 19–24 is normal, BMI 24–28 is overweight and BMI ≥28 is obese.

All laboratory measurements were carried out at the local hospitals where the visits took place. For data collection and quality control, all the data were recorded in the approved case report form and entered into a web‐based electronic data capture system designed by VitalStrategic Research Institute (VSRI, Shanghai, China).

### Statistical analysis

Descriptive statistics were used to characterize the data in the study, including calculations of means and standard deviations. The frequency and percentages (based on the non‐missing sample size) of observed levels were reported for all categorical measures. Comparisons were statistically analyzed using anova and χ^2^‐tests. The primary outcome was the overall proportion of patients reaching HbA1c <7.0% at the end of 1‐year follow up. The generalized estimating equation was applied for the multiple analyses of primary end‐points to assess the relative risks (RR) and 95% confidence interval (CI). The selections of independent variables were determined by both clinical experiences and factor contribution. The generalized estimating equation model was used to evaluate the influential factors associated with the time to the changes for the hypoglycemic treatment pattern. The models included the three time‐dependent variables: hypoglycemic treatment paradigm, study visit and the reason of treatment change; and were adjusted for pre‐selected baseline characteristics: patient's blood glucose level, blood pressure and blood lipid level, adequate HbA1c control, sex, age, education, insurance type, family income, health behaviors and so on. A *P*‐value <0.05 for the two‐tailed test was considered as statistically significant. Statistical analyses were carried out using SAS version 9.3 (SAS Institute, Cary, NC, USA; Appendix S1).

## Results

### Characteristics of newly diagnosed type 2 diabetes patients

A total of 5,985 patients were recruited from 81 hospitals across six geographic regions of China, but the data of 215 patients from two hospitals were removed from the final analyses due to the failure of passing the study audits. Eventually, 5,770 patients from 79 hospitals were included in this report (Table S1). The average age of these patients was 55.7 ± 12.6 years, 54.2% were men and the mean BMI was 25.0 ± 3.4 kg/m^2^. The mean HbA1c of patients was 8.4 ± 2.5% at baseline, with 36.8% of them reaching the target of HbA1c <7.0%. A total of 37.3% of the patients had hypertension, and 46.3% of them had dyslipidemia at baseline. Proportions of patients from tier 1, tier 2 and tier 3 hospitals were 23.6, 27.3 and 49.0%, respectively (Table 1). Baseline demographics under hypoglycemia treatment patterns are shown in Tables 2, S2 and S3. The compliance of patients is also shown in Table 2. Dipeptidyl peptidase‐4 inhibitors were included in “Others” in Table 2.

**Table 1:** Baseline characteristics of newly diagnosed patients with type 2 diabetes in China

Characteristics	Total
All patients (*n*)	5,770
Age, years (mean ± SD)	55.7 ± 12.6
Sex, *n* (%)	
Male	3,130 (54.2%)
Female	2,640 (45.8%)
Smoking status, *n* (%)	
None	3,902 (67.6%)
Current	1,271 (22.0%)
Previous	505 (8.8%)
Passive	92 (1.6%)
Drinking status, *n* (%)	
None	4,860 (84.2%)
Current	619 (10.7%)
Previous	291 (5.0%)
Physical activities, *n* (%)	
No exercises	1,348 (23.4%)
≤3 times/week	2,406 (41.7%)
>3 times/week	2,016 (34.9%)
Medicine compliance, *n* (%)	
Yes	5,278 (91.5%)
No	492 (8.5%)
BMI, kg/m^2^ (mean ± SD)	25.0 ± 3.4
BMI category, *n* (%)	
<24 kg/m^2^	2,249 (39.0%)
24 to <28 kg/m^2^	2,544 (44.1%)
≥28 kg/m^2^	977 (16.9%)
Family history of diabetes, *n* (%)	
Yes	1,628 (28.2)
No	3,962 (68.7)
Unknown	180 (3.1)
Family history of cardiovascular disease, *n* (%)	
No	4,429 (76.8)
Yes	1,067 (18.5)
Unknown	274 (4.7)
Hypertension, *n* (%)	2,152 (37.3%)
Dyslipidemia, *n* (%)	2,670 (46.3%)
Region	
North	573 (9.9)
South	915 (15.9)
East	782 (13.6)
Southwest	1,503 (26.0)
Northeast	856 (14.8)
Northwest	1,141 (19.8)
Hospital tier	
1st tier	1,364 (23.6)
2nd tier	1,577 (27.3)
3rd tier	2,829 (49.0)
Comorbidities	
Diabetes only	2,090 (36.2)
Diabetes + hypertension	1,010 (17.5)
Diabetes + dyslipidemia	1,528 (26.5)
Diabetes + hypertension + dyslipidemia	1,142 (19.8)
HbA1c %, mmol/mol (mean ± SD)	
Total	8.4 ± 2.5(68 ± 19)
SBP, mmHg (mean ± SD)	
Total	129 ± 14
DBP, mmHg (mean ± SD)	
Total	79 ± 9
T‐CHO, mmol/L (mean ± SD)	5.0 ± 1.3
HDL‐C, mmol/L (mean ± SD)	1.2 ± 0.4
LDL‐C, mmol/L (mean ± SD)	2.9 ± 1.0
TG, mmol/L (mean ± SD)	2.4 ± 11.6

**Table 2:** Baseline characteristics under hypoglycemic treatment patterns in newly diagnosed type 2 diabetes patients in China

Patient groups	Total *n* (%)	No OAD or insulin	Only herbal	OAD only			Insulin use				*P*‐value
		*n* (%)	*n* (%)	1 OAD *n* (%)	2 OAD *n* (%)	≥3 OAD *n* (%)	Insulin only *n* (%)	Insulin + 1 OAD *n* (%)	Insulin + 2 OAD *n* (%)	Insulin + ≥3 OAD *n* (%)	
Total	5,770 (100.0)	2,527 (43.8)	48 (0.8)	1,308 (22.7)	742 (12.9)	122 (2.1)	559 (9.7)	318 (5.5)	122 (2.1)	24 (0.4)	
HbA1c	8.4 ± 2.5	8.3 ± 2.4	7.0 ± 1.6	7.4 ± 1.9	8.3 ± 2.3	8.8 ± 2.6	9.8 ± 2.7	10.0 ± 2.7	10.2 ± 2.7	10.4 ± 2.7	<0.0001
Sex (*n*)											
Male	3,130 (54.2)	1,411 (55.8)	21 (43.8)	611 (46.7)	379 (51.1)	54 (44.3)	360 (64.4)	202 (63.5)	72 (59.0)	20 (83.3)	<0.0001
Female	2,640 (45.8)	1,116 (44.2)	27 (56.3)	697 (53.3)	363 (48.9)	68 (55.7)	199 (35.6)	116 (36.5)	50 (41.0)	4 (16.7)	
Age group (years)											
20 to <65	4,408 (76.4)	1,946 (77.0)	34 (70.8)	942 (72.0)	558 (75.2)	102 (83.6)	443 (79.2)	263 (82.7)	100 (82.0)	20 (83.3)	<0.001
≥65	1,362 (23.6)	581 (23.0)	14 (29.2)	366 (28.0)	184 (24.8)	20 (16.4)	116 (20.8)	55 (17.3)	22 (18.0)	4 (16.7)	
Smoking											
Never	3,902 (67.6)	1,686 (66.7)	33 (68.8)	959 (73.3)	499 (67.3)	93 (76.2)	342 (61.2)	201 (63.2)	78 (63.9)	11 (45.8)	<0.0001
Current	1,271 (22.0)	606 (24.0)	10 (20.8)	217 (16.6)	152 (20.5)	17 (13.9)	138 (24.7)	86 (27.0)	37 (30.3)	8 (33.3)	
Previous	505 (8.8)	199 (7.9)	2 (4.2)	102 (7.8)	77 (10.4)	10 (8.2)	72 (12.9)	31 (9.7)	7 (5.7)	5 (20.8)	
Passive	92 (1.6)	36 (1.4)	3 (6.3)	30 (2.3)	14 (1.9)	2 (1.6)	7 (1.3)	0 (0.0)	0 (0.0)	0 (0.0)	
Drinking											
Never	4,867 (84.4)	2,110 (83.5)	40 (83.3)	1,145 (87.5)	633 (85.3)	112 (91.8)	454 (81.2)	260 (81.8)	97 (79.5)	16 (66.7)	<0.0001
Current	620 (10.7)	324 (12.8)	6 (12.5)	105 (8.0)	58 (7.8)	8 (6.6)	57 (10.2)	37 (11.6)	18 (14.8)	7 (29.2)	
Previous	283 (4.9)	93 (3.7)	2 (4.2)	58 (4.4)	51 (6.9)	2 (1.6)	48 (8.6)	21 (6.6)	7 (5.7)	1 (4.2)	
Physical activities											
No exercise	1,348 (23.4)	628 (24.9)	11 (22.9)	259 (19.8)	174 (23.5)	29 (23.8)	137 (24.5)	69 (21.7)	33 (27.0)	8 (33.3)	<0.05
Occasional exercise†	2,406 (41.7)	1,049 (41.5)	14 (29.2)	550 (42.0)	299 (40.3)	51 (41.8)	232 (41.5)	145 (45.6)	58 (47.5)	8 (33.3)	
Regular exercise	2,016 (34.9)	850 (33.6)	23 (47.9)	499 (38.1)	269 (36.3)	42 (34.4)	190 (34.0)	104 (32.7)	31 (25.4)	8 (33.3)	
Medication compliance											
Yes	5,278 (91.5)	2,158 (85.4)	44 (91.7)	1,250 (95.6)	721 (97.2)	113 (92.6)	545 (97.5)	304 (95.6)	120 (98.4)	23 (95.8)	<0.0001
No	492 (8.5)	369 (14.6)	4 (8.3)	58 (4.4)	21 (2.8)	9 (7.4)	14 (2.5)	14 (4.4)	2 (1.6)	1 (4.2)	
BMI											
<24 kg/m^2^	2,249 (39.0)	958 (37.9)	17 (35.4)	510 (39.0)	262 (35.3)	46 (37.7)	263 (47.0)	142 (44.7)	42 (34.4)	9 (37.5)	<0.01
24.0 to < 28 kg/m^2^	2,544 (44.1)	1,132 (44.8)	26 (54.2)	552 (42.2)	353 (47.6)	59 (48.4)	221 (39.5)	126 (39.6)	64 (52.5)	11 (45.8)	
≥28 kg/m^2^	977 (16.9)	437 (17.3)	5 (10.4)	246 (18.8)	127 (17.1)	17 (13.9)	75 (13.4)	50 (15.7)	16 (13.1)	4 (16.7)	
Region											
North	573 (9.9)	272 (10.8)	3 (6.3)	163 (12.5)	79 (10.6)	13 (10.7)	13 (2.3)	16 (5.0)	12 (9.8)	2 (8.3)	<0.0001
South	915 (15.9)	391 (15.5)	8 (16.7)	230 (17.6)	135 (18.2)	30 (24.6)	70 (12.5)	35 (11.0)	12 (9.8)	4 (16.7)	
East	782 (13.6)	298 (11.8)	5 (10.4)	229 (17.5)	118 (15.9)	18 (14.8)	48 (8.6)	38 (11.9)	22 (18.0)	6 (25.0)	
Southwest	1,503 (26.0)	607 (24.0)	6 (12.5)	278 (21.3)	206 (27.8)	43 (35.2)	227 (40.6)	92 (28.9)	37 (30.3)	7 (29.2)	
Northeast	856 (14.8)	489 (19.4)	4 (8.3)	123 (9.4)	51 (6.9)	10 (8.2)	109 (19.5)	50 (15.7)	18 (14.8)	2 (8.3)	
Northwest	1,141 (19.8)	470 (18.6)	22 (45.8)	285 (21.8)	153 (20.6)	8 (6.6)	92 (16.5)	87 (27.4)	21 (17.2)	3 (12.5)	
Hospital tier											
1st tier	1,364 (23.6)	600 (23.7)	29 (60.4)	450 (34.4)	180 (24.3)	21 (17.2)	51 (9.1)	26 (8.2)	4 (3.3)	3 (12.5)	<0.0001
2nd tier	1,577 (27.3)	699 (27.7)	10 (20.8)	343 (26.2)	223 (30.1)	44 (36.1)	140 (25.0)	69 (21.7)	42 (34.4)	7 (29.2)	
3rd tier	2,829 (49.0)	1,228 (48.6)	9 (18.8)	515 (39.4)	339 (45.7)	57 (46.7)	368 (65.8)	223 (70.1)	76 (62.3)	14 (58.3)	

^†^Definition of occasional exercise is that patients exercise <150 min per week, as the recommendation of exercise by the Chinese Diabetes Guideline is that type 2 diabetes patients should have at least 150 min of exercise per week. BMI, body mass index; HbA1c, glycated hemoglobin A1c; OAD, oral antidiabetic drug.

### Current hypoglycemic treatment paradigms

At baseline, 43.8% of the patients were on diet and exercise alone, and 0.8% of them were taking herbal medicine only. Of the patients taking hypoglycemic drugs (55.4%), 37.7% of the patients took OHAs only (22.7% with one OHA, 12.9% with two OHAs and 2.1% with more than two OHAs, respectively), and 17.7% of them received insulin treatment. Metformin use was the most common (12.4%) in one OHA treatment, metformin with sulfonylurea use was the most common (4.6%) in two OHAs treatment and metformin with insulin use was the most common (2.9%) in insulin with one OHA treatment (Table 3). The associations between baseline risk factors and baseline hypoglycemic medication are also provided in Table S4. Among those patients who took hypoglycemic drugs, 54.6% of them changed their antidiabetes treatment (ADT) at baseline, of which, 16.5% had dosage adjustment, 4.1% switched hypoglycemic drug, 0.9% added new drugs and 33.1% discontinued hypoglycemic drugs (Table S5).

**Table 3:** Hypoglycemic treatment patterns of newly diagnosed type 2 diabetes patients in China

Hypoglycemic group	Baseline	3 months	6 months	9 months	12 months
	*n* (%)				
Total	5,770	4,989	4,817	4,658	4,562
No OHA or insulin					
Total	2,575 (44.6)	1,051 (21.1)	1,035 (21.5)	1,022 (21.9)	1,089 (23.9)
Diet and exercises	2,527 (43.8)	995 (19.9)	982 (20.4)	977 (21.0)	1,040 (22.8)
Herbal medicine	48 (0.8)	56 (1.1)	53 (1.1)	45 (1.0)	49 (1.1)
One OHA, no insulin					
Total	1,308 (22.7)	1,657 (33.2)	1,636 (34.0)	1,585 (34.0)	1,532 (33.6)
MF	713 (12.4)	893 (17.9)	901 (18.7)	865 (18.6)	836 (18.3)
AGI	236 (4.1)	299 (6.0)	287 (6.0)	277 (5.9)	284 (6.2)
SU	220 (3.8)	251 (5.0)	258 (5.4)	261 (5.6)	242 (5.3)
Others†	139 (2.4)	214 (4.3)	190 (3.9)	182 (3.9)	170 (3.7)
Two OHAs, no insulin					
Total	742 (12.9)	955 (19.1)	941 (19.5)	934 (20.1)	902 (19.8)
MF + SU	268 (4.6)	343 (6.9)	361 (7.5)	368 (7.9)	341 (7.5)
MF + AGI	138 (2.4)	174 (3.5)	178 (3.7)	165 (3.5)	166 (3.6)
AGI + SU	99 (1.7)	125 (2.5)	121 (2.5)	128 (2.7)	126 (2.8)
MF + glinides	93 (1.6)	109 (2.2)	92 (1.9)	85 (1.8)	95 (2.1)
MF + others	77 (1.3)	112 (2.2)	109 (2.3)	112 (2.4)	101 (2.2)
Any combinations except listed above	67 (1.2)	92 (1.8)	80 (1.7)	76 (1.6)	73 (1.6)
More than two OHAs, no insulin					
Total	122 (2.1)	181 (3.6)	191 (3.8)	165 (3.5)	140 (3.1)
Insulin only, no OHA					
Total	559 (9.7)	557 (11.2)	481 (9.6)	480 (10.3)	463 (10.1)
Insulin + one OHA					
Total	318 (5.5)	384 (7.7)	353 (7.1)	315 (6.8)	305 (6.7)
MF	167 (2.9)	189 (3.8)	174 (3.5)	151 (3.2)	139 (3.0)
AGI	97 (1.7)	120 (2.4)	111 (2.2)	107 (2.3)	106 (2.3)
Others	54 (0.9)	75 (1.5)	68 (1.4)	57 (1.2)	60 (1.3)
Insulin + two OHAs					
Total	122 (2.1)	167 (3.3)	155 (3.2)	133 (2.9)	111 (2.4)
Insulin + more than two OHAs					
Total	24 (0.4)	37 (0.7)	25 (0.5)	24 (0.5)	20 (0.4)

^†^Dipeptidyl peptidase‐4 inhibitors were included in others. AGI, α‐glucosidase inhibitor; MF, metformin; OHA, oral hypoglycemic agent; SU, sulfonylureas.

### Glycemic control

**Figure 1:**
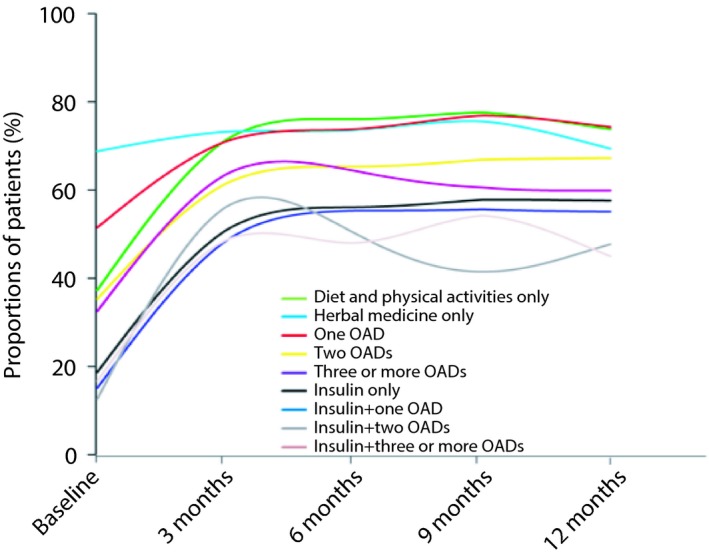
Proportion of newly diagnosed type 2 diabetes patients reaching the target of glycated hemoglobin A1c <7.0% under hypoglycemic treatment patterns at baseline and during the 12 months of follow up. OADs, oral antidiabetic drugs.

The mean HbA1c of the patients was 6.7 ± 1.2% at 12 months, with a 1.5 ± 2.4% decrease from baseline (*P *<* *0.0001), and 68.5% of the patients reaching HbA1c <7.0%. A total of 74.3% of the patients taking one OHA reached the target of HbA1c <7.0% at 12 months, which was the highest among variable treatments, with 7.4 ± 1.9% of HbA1c levels at baseline and 1.1 ± 2.1% decrease from baseline (*P *<* *0.0001). A total of 45.0% of patients taking insulin with more than two OHAs reached the target at 12 months, which was the lowest, with 10.5 ± 2.7% of HbA1c at baseline and 3.1 ± 3.1% reduction from baseline (Figure 1; Table S3). In Table S6, the mean levels of HbA1c (%) stratified by patient characteristics were also provided.

### Determinants of change in HbA1c

#### Treatment patterns

**Figure 2:**
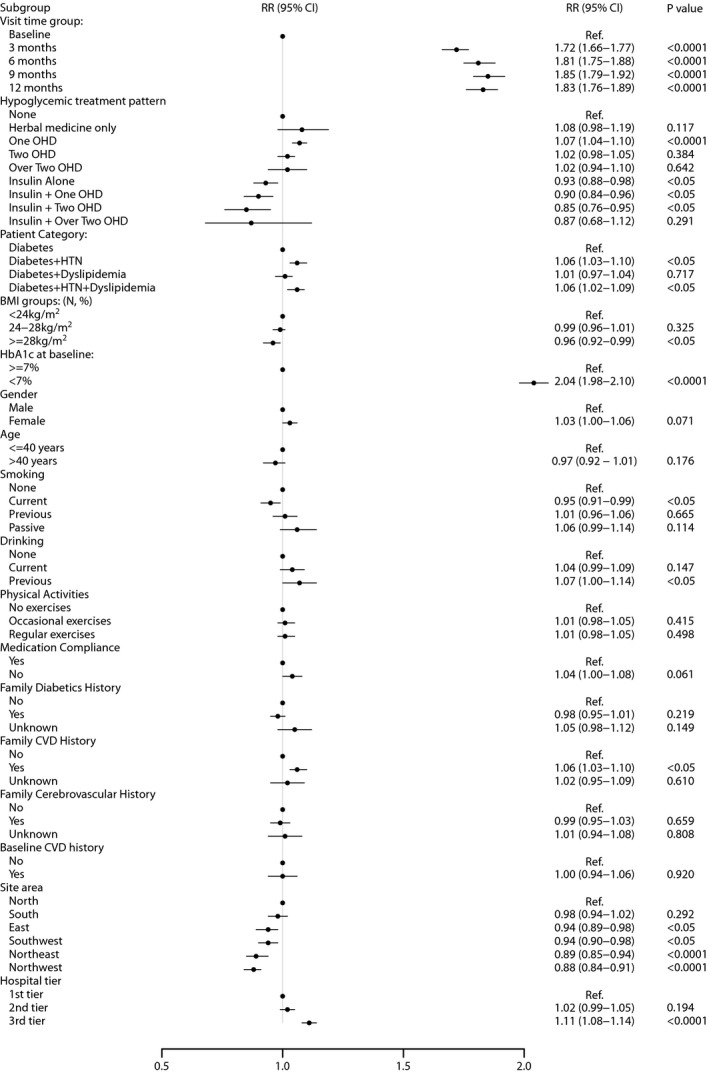
Multivariate analysis of hypoglycemic treatment on glycated hemoglobin A1c control and other associated factors with glycated hemoglobin A1c control in newly diagnosed type 2 diabetes patients in China. BMI, body mass index; CI, confidence interval; CVD, cardiovascular disease; HTN, hypertension; OHD, oral hypoglycemic drug; RR, relative risk.

Multiple analysis showed that compared with the patients without any hypoglycemic medications, the patients with one OHA had a significantly increased possibility of reaching adequate glycemic control (RR 1.07; *P *<* *0.001), whereas the patients with insulin alone (RR 0.93; *P *=* *0.01), insulin plus one OHA (RR 0.90; *P *=* *0.003) and insulin plus two OHAs (RR 0.85; *P *=* *0.005) had lower possibilities of reaching the target (Figure 2).

#### Comorbidities

The type 2 diabetes patients with hypertension (RR 1.07; *P *<* *0.0001), or with hypertension and dyslipidemia (RR 1.06; *P *=* *0.0008) had higher possibilities of reaching adequate glycemic controls compared with type 2 diabetes patients with neither hypertension nor dyslipidemia. A stratified analysis according to type 2 patients with hypertension or with dyslipidemia, or both, also showed a significant difference in the proportions of reaching the treatment target in patients with different comorbidities (Table S7).

#### Associated baseline characteristics

Obese patients (BMI ≥28 kg/m^2^; RR 0.96; *P *=* *0.01) and active smokers (RR 0.95; *P *=* *0.008) tended to have a lower possibility of reaching adequate controls, whereas the patients with a family history of cardiovascular disease (RR 1.06; *P *=* *0.001) or who achieved HbA1c <7.0% (53 mmol/mol) at baseline (RR 2.04; *P *<* *0.0001) had higher possibilities of reaching adequate glycemic controls.

#### Regions and hospitals

The glycemic control differences also existed across six geographic recruitment regions (*P *<* *0.0001) and different tiers of hospitals (*P *<* *0.0001; Figure 2). A stratified analysis according to tier 1, tier 2 and tier 3 hospitals showed the difference in the proportions of patients reaching the treatment target in different tiers of hospitals (*P *<* *0.0001; Table S7).

### Treatment modifications and reasons

After 12 months of follow up, 23.9% of the patients were on diet and exercise alone or with herbal medicine, 56.5% of them took OHAs only, and 19.6% received insulin treatment. A total of 18.3% of the patients took metformin as monotherapy, and 7.5% had metformin with sulfonylurea as combination therapy (Table 2). The proportion of patients that modified their therapeutic regimens decreased significantly to 15.7% (4.6% with dosage adjustment, 1.1% with drug switching, 4.1% with new drugs addition and 5.9% with treatment discontinuation; *P* < 0.0001; Table S5).

The four most commonly reported reasons for treatment alterations were patient's personal choice (27.8%), poor efficacy (24.3%), adverse effects (1.7%) and economic factors (1.1%) at baseline. The proportions of patients who reported the aforementioned reasons all decreased significantly after 12 months (*P* < 0.0001; Table S5).

### Associated factors with treatment alterations

#### Time of follow up

Multivariate generalized estimating equation models suggested that the risk of treatment alterations decreased gradually and significantly on ADT dosage adjustment (*P* < 0.0001), switching ADT (*P* < 0.0001), discontinuing ADT (*P* < 0.0001) and any ADT changes (*P* < 0.0001) during the time of follow up, whereas a linear trend of increasing risk of adding new ADT was observed over time (*P* < 0.0001; Table S8).

#### Treatment patterns

Compared with patients without any drugs, those taking two or more OHAs and those taking insulin in combination with two or more OHAs were less likely to adjust ADT dosage (*P* = 0.002 and 0.02, respectively). Compared with those taking drugs, patients taking one OHA were less likely to discontinue ADT (RR 0.67, *P* < 0.0001). Compared with patients not taking drugs, those taking either ADT were less likely to add new ADT agents (*P* = 0.0005) or make any ADT changes (*P* < 0.0001; Table S8).

#### Patient‐reported reasons

Among patient‐reported reasons for treatment alterations, the economic factors were the most significant reason associated with ADT switching (*P* < 0.0001) and new ADT addition (*P* < 0.0001). The most important reason for dose adjustment (*P* < 0.0001) and any ADT changes (*P* < 0.0001) was poor efficacy, whereas patient's personal choices were the main reason for ADT discontinuation (*P* < 0.0001; Table S8).

#### Regions and tier of hospitals

Significant regional differences were also found on hypoglycemic treatment modifications, and patients from different tiers of hospitals had differences in treatment alterations (Table S8).

#### Baseline patient characteristics

However, the majority of patient characteristics, such as patient category (diabetes alone, diabetes with hypertension or dyslipidemia), baseline HbA1c level, sex, age, education, BMI and so on, were not significantly associated with any kind of treatment alterations (Table S9).

## Discussion

The CCMR‐NEW2D study was a pioneering large‐scale prospective cohort study, to investigate clinical outcomes in newly diagnosed type 2 diabetes patients in China, and the hypoglycemic treatment pattern and evolution during the first year after diagnosis. Overall, the mean HbA1c of these patients decreased significantly from 8.4 ± 2.5% to 6.7 ± 1.2%, and 68.5% of them met the target of HbA1c <7% by the end of 12 months. Compared with the earlier national surveys that reported 39.7% of patients meeting the target in a population‐based study^3^ and 31.78–47.7% of the patients reaching glycemic control in hospital‐based studies^9^, ^12^, ^13^, the newly diagnosed type 2 diabetes patients in the present study achieved similar glycemic control at baseline, but better control after 12 months. Multivariate analysis showed high associations between follow‐up visits and glycemic control, suggesting that newly diagnosed patients who were willing to return for follow‐up visits every 3 months might improve their glycemic control. Furthermore, patients with obesity, current smoking, baseline HbA1c ≥7% or living in east, southwest, northeast and northwest parts of China were less likely to obtain their treatment targets, some of which could be explained in previously published data^12^, ^15^, ^16^, ^17^. However, regional differences were rarely reported.

The present study also outlined the hypoglycemic treatment patterns and evolutions in newly diagnosed type 2 diabetes patients in China. The proportions of patients without hypoglycemic medications, with OHAs and with insulin were 44.6, 37.7 and 17.7%, respectively, at baseline, and were 23.9, 56.5 and 19.6%, respectively, after 12 months. A previous hospital‐based study^9^ of type 2 diabetes patients showed that 55% of the patients took OHAs and 35.7% took insulin, just 9.3% did not take medications. In another Hong Kong Diabetes Registry^10^, 7.9% type 2 diabetes patients were receiving dietary treatment only, 52.9% were taking OHAs and 39.2% were receiving insulin therapy. Differences between previous results and the present result might indicate that the newly diagnosed type 2 diabetes patients were more willing not to take hypoglycemic medications or the physicians preferred not to prescribe ADT at the beginning of diagnosis, although the patients’ baseline HbA1c levels were >7%. Among those taking hypoglycemic drugs in the present study, the proportion of patients receiving metformin increased from approximately 25.2% at baseline to 36.7% after 12 months, suggesting that metformin was the most common agent (~66.2%) taken by Chinese newly diagnosed type 2 diabetes patients, similar to that reported in USA between 1998 and 2009^18^.

Furthermore, we found 17.7% of the patients at baseline and 19.6% at 12 months received insulin treatment. As recommended by the guidelines of the China Diabetes Society^7^, ^8^, for newly diagnosed type 2 diabetes patients with HbA1c >9.0%, short‐term intensive insulin therapy can be implemented. The updated guideline of the American Diabetes Association^5^, also suggested considering initiating insulin therapy for newly diagnosed type 2 diabetes patients with HbA1c ≥10%. Intensive insulin therapy as an option for Chinese newly diagnosed type 2 diabetes patients also showed efficacy^19^, ^20^. In the present study, the baseline levels of HbA1c in patients receiving insulin treatment were significantly higher than others, indicating these patients had poor glycemic control. The results from multivariate analysis suggesting that patients receiving insulin treatment had a lower possibility of reaching HbA1c target compared with no treatment also confirmed this opinion.

In the present study, more than half of the medication therapy underwent changes, and the most common reasons for treatment alterations were poor efficacy, patient's personal choices, adverse effects and economic reasons. With the increase of patients meeting the target of glycemic control, the proportions of patients with treatment modifications gradually decreased. It was suggested that a patient‐centered approach should be used, and considerations for the choice of pharmacological agents should include drug efficacy, potential side‐effects, cost and patient preferences^5^, ^6^, ^7^, ^8^, which was also shown by the present study of Chinese patients. We also found that treatment modification was associated with baseline treatment patterns. Compared with patients not taking any ADT drugs, the patients taking either ADT were less likely to make any ADT changes, suggesting that although nearly half of the patients did not take medications at the time of diagnosis, with the progression of disease, they need to change treatment patterns for better glycemic control.

Gaps were observed between real‐world diabetes management and the recommendations for treatment strategy in the present study with newly diagnosed patients. As previously shown^21^, ^22^, ^23^, ^24^, ^25^, there were many challenges for implementing evidence into practice in relation to diabetes prevention, treatment and management across the world. In the achievement of recommended targets, in the adherence to guidelines and in the adherence to recommended treatments, we should search for solutions for people with diabetes, especially for newly diagnosed patients.

As an observational cohort study, there were some limitations. First, no further correction was carried out for the value of HbA1c in the CCMR‐NEW2D study. The results of laboratory tests from variable hospitals were accepted, taking real‐world evidence and study cost into consideration. Fortunately, in recent years, a series of industry standards have been implemented, thus the reference difference among individual laboratories has become small^26^, ^27^. Second, as an observational study with 12 months of follow up, this duration of follow up is not enough to give us comprehensive answers about the clinical outcomes, such as macrovascular and microvascular complications, associated with the hypoglycemic treatment patterns. Third, the influence of more factors, such as eating habits, patients’ professions and the safety of long‐term use of medicines, was not collected. Thus, a multiple‐year longitudinal cohort study will be required. Furthermore, as a real‐world observational study, selection bias could not be avoided.

The present longitudinal cohort study provides valuable information on current hypoglycemic treatment in newly diagnosed type 2 diabetes patients in China, outlines the glycemic control, hypoglycemic treatment patterns and alterations with the associated factors in these patients, and reveals gaps between real‐world treatment patterns and clinical guidelines.

## Disclosure

LJ has received fees for lecture presentations from AstraZeneca, Merck, Novartis, Lilly, Roche, Sanofi‐Aventis and Takeda. LJ has received consulting fees from companies including AstraZeneca, Merck, Novartis, Lilly, Roche, Sanofi‐Aventis and Takeda. LJ has received grants/research support from AstraZeneca, Bristol‐Myers Squibb, Merck, Novartis, and Sanofi‐Aventis. The other authors declare no conflict of interest.

## Supporting information


**Table S1 ** Distribution of hospitals across China from which patients were recruited.
**Table S2** Baseline demographics including education, insurance, family income and comorbidities in newly diagnosed type 2 diabetes patients in China.
**Table S3** Glycemic control in newly diagnosed type 2 diabetes patients in China.
**Table S4** Associations between baseline risk factors and baseline hypoglycemic medication.
**Table S5** Modifications and reasons of hypoglycemic treatment pattern over time.
**Table S6** Mean Levels of glycated hemoglobin A1c (%) by patient characteristics.
**Table S7** Baseline characteristics and proportions of treatment target stratified by comorbidities and hospitals.
**Table S8** Multivariate analyses of risk factors on antidiabetes treatment changes.
**Table S9** Multivariate analysis for risk factors of patient characteristics on antidiabetes treatment changes.
**Table S10** Hospitals included in the present study for patient recruitment.
**Appendix S1** eProtocol.
**Appendix S2** eChecklist.
